# Crystal structure of the coordination polymer *catena*-poly[[[(acetonitrile-κ*N*)copper(I)]-μ_3_-1,3-dithiolane-κ^3^ *S*:*S*:*S*′] hexafluoridophosphate]

**DOI:** 10.1107/S205698901901627X

**Published:** 2020-01-01

**Authors:** Lena Knauer, Michael Knorr, Lydie Viau, Carsten Strohmann

**Affiliations:** a Anorganische Chemie, Technische Universität Dortmund, Otto-Hahn-Strasse 6, D-44227 Dortmund, Germany; b Institut UTINAM UMR 6213 CNRS, Université Bourgogne Franche-Comté, 16 Route de Gray, 25030 Besançon Cedex, France

**Keywords:** crystal structure, copper complex, coordination polymer, thio­ether, C—H⋯F hydrogen bonding

## Abstract

The polymeric title compound, [Cu_2_(C_2_H_3_N)_2_(C_3_H_6_S_2_)_2_](PF_6_)_2_, represents an example of a one-dimensional coordination polymer resulting from the reaction of [Cu(MeCN)_4_][PF_6_] with 1,3-di­thiol­ane. The cationic one-dimensional ribbon consists of two copper(I) centers each ligated by one aceto­nitrile mol­ecule and inter­connected through two bridging 1,3-di­thiol­ane ligands. One S-donor site of each ligand is κ^1^-bound to Cu, whereas the second S atom acts as a four-electron donor, bridging two Cu atoms in a κ^4^-bonding mode. The positive charge of each copper cation is compensated for by a hexa­fluorido­phosphate counter-ion. In the crystal, the polymer chains are linked by a series of C—H⋯F hydrogen bonds, forming a supra­molecular framework.

## Abstract 2

The title one-dimensional ribbon-like coordination polymer results from the reaction of [Cu(MeCN)_4_][PF_6_] with 1,3-di­thiol­ane.

## Chemical context   

The five-membered heterocyclic ligand tetra­hydro­thio­phene (THT) is known to form a great variety of mol­ecular complexes and coordination polymers (CPs) with various transition metals. Notably, for the soft coinage metal ions copper(I), silver(I) and gold(I), numerous structurally characterized examples coordinated by terminal or bridging THT ligands have been documented (Ahrland *et al.*, 1993 ▸; Dembo *et al.*, 2010 ▸; Norén & Oskarsson, 1985 ▸; Mälger *et al.*, 1992 ▸; Usón *et al.*, 1984 ▸). Even mixed-valence (Cu^I^–Cu^II^) compounds such as polymeric penta-μ-chloro-tris-μ-tetra­hydro­thio­phene­tetra­copper(I,II) have been prepared (Ainscough *et al.*, 1985 ▸). In the case of the five-membered heterocycle 1,2-di­thiol­ane, in which one CH_2_ unit is replaced by a second sulfur atom, there is one report on its coordination to Hg_2_(NO_3_)_2_ yielding the Hg^I^ adduct 1,2-di­thiol­ane·Hg_2_(NO_3_)_2_ (Brodersen & Rölz, 1977 ▸). Furthermore, the dinuclear organometallic species [η^5^-CpMn(CO)_2_(μ_2_-1,2-di­thiol­ane)]_2_ has been characterized crystallographically (Braunwarth *et al.*, 1991 ▸). The fluxional complexes [*M*(CO)_5_(1,3-di­thiol­ane)] (*M* = Cr, Mo, W) ligated by the isomeric heterocycle 1,3-di­thiol­ane (1,3-di­thia­cyclo­penta­ne) have been investigated by NMR spectroscopy (Abel *et al.*, 1990 ▸).

In a comparative study with respect to our previous work on the coordination chemistry of the open-chain di­thio­ether analogues *R*S-CH_2_-S*R* (Chaabéne *et al.*, 2016 ▸; Knorr *et al.*, 2014 ▸; Peindy *et al.*, 2007 ▸) and in part to fill the gap between the versatile coordination chemistry of THT (see above) and the almost unexplored coordination chemistry of 1,3-di­thiol­ane, we recently described in detail the construction and structural features of mol­ecular clusters and coordination networks, with dimensionalities varying from 0D–2D by reacting 1,3-di­thiol­ane and its ferrocenyl derivative substituted at the 2-position with Cu*X* salts (*X* = Cl, Br, I) (Raghuvanshi *et al.*, 2017 ▸). However, surprisingly, a survey of the Cambridge Structural Database (Groom *et al.*, 2016 ▸), reveals that apart from our Cu*X*–1,3-di­thiol­ane compounds, no other unsubstituted 1,3-di­thiol­ane complexes have been structurally characterized. We have now extended our project on the coordination chemistry of this cyclic di­thio­ether using [Cu(MeCN)_4_][PF_6_] as reactant to obtain the title polymeric ionic salt-like material, which could be inter­esting for electrochemical investigations.
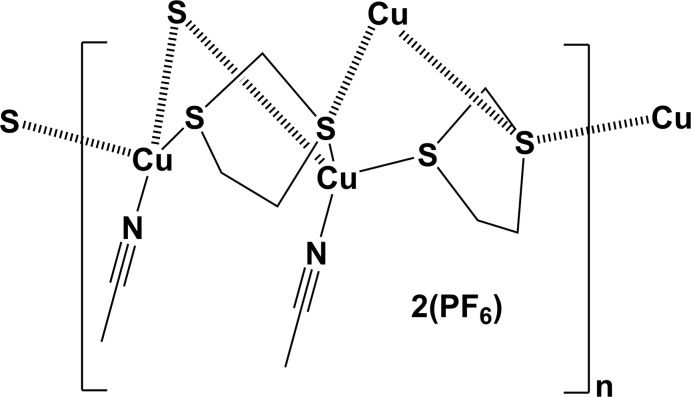


## Structural commentary   

We have previously described (Raghuvanshi *et al.*, 2017 ▸), the structural features of the ribbon-like structures of compounds [{Cu(μ_2_-Br)}(μ_2_-**L1**)]_*n*_ and [{Cu(μ_2_-Cl)}(μ_2_-**L1**)]_*n*_, formed upon treatment of CuBr and CuCl with 1,3-di­thiol­ane (**L1**). The title complex salt, a ribbon of composition [Cu(1,3-di­thiane)(MeCN)]_*n*_^+^ (**CP1**) also results from the reaction of [Cu(MeCN)_4_][PF_6_] with **L1**, but its architecture is quite different.

**Figure 1:**
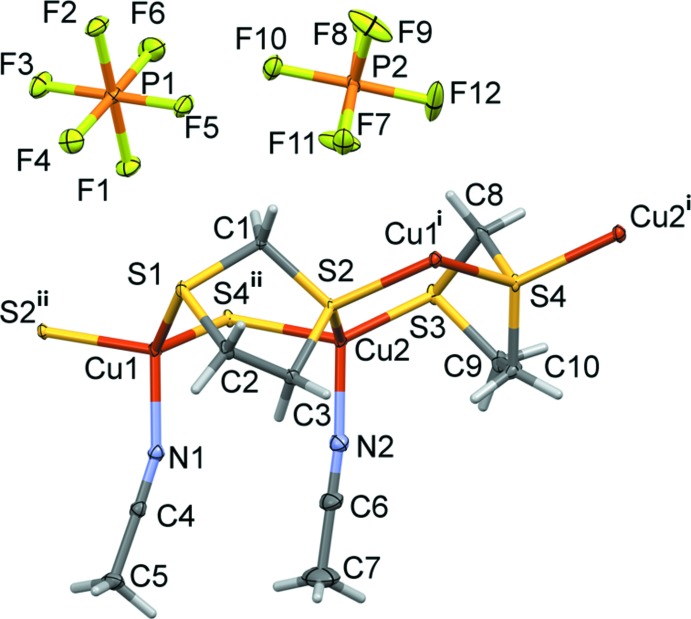
A view of the asymmetric unit of the title compound, with atom labelling [symmetry codes: (i) *x* − 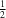, −*y* + 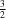, *z*; (ii) *x* + 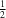, −*y* + 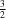, *z*]. Displacement ellipsoids are drawn at the 30% probability level.

**Figure 2:**
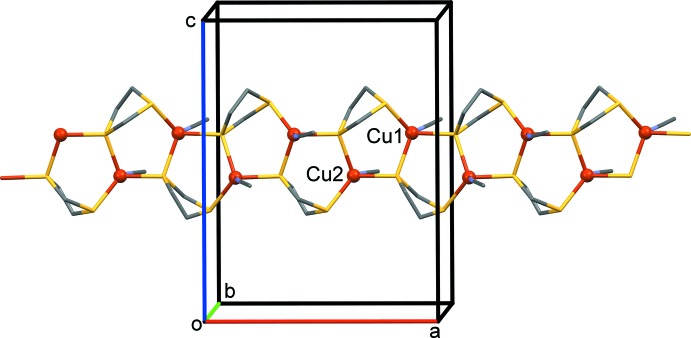
A partial view along the *b* axis of the crystal packing of the title compound. For clarity, the H atoms and the PF_6_^−^ anions have been omitted.

The mol­ecular structure of the asymmetric unit of the title complex is illustrated in Fig. 1 ▸, and selected bond lengths and bond angles are given in Table 1 ▸. The ribbon-like structure is built upon individual Cu^I^ atoms, each ligated by a datively bound MeCN ligand and inter­connected to the neighbouring metal centers by two bridging di­thiol­ane ligands (Fig. 2 ▸). Overall, the architecture of **CP1** is quite reminiscent of that of the 1D polymeric tetra­fluorido­borate salt [Cu(1,3-di­thiane)(MeCN)]_*n*_^+^ (Knaust & Keller, 2003 ▸). Nevertheless, there is one difference. Whereas the asymmetric unit of the latter salt (crystallizing in the ortho­rhom­bic Sohncke space group *P*2_1_2_1_2_1_) contains three unique copper(I) centers, that of **CP1** (crystallizing in the ortho­rhom­bic non-centrosymmetric space group *Pna*2_1_) contains only two unique Cu^I^ atoms. Each displays a CuNS_3_ four-coordinate environment; see Table 1 ▸ [*L*—Cu—*L* angles: 99.97 (7) to 119.47 (11)° for Cu1, and 99.29 (11) to 118.69 (4)° for Cu2]. The τ_4_ descriptor for fourfold coordination is = 0.89 for both atoms Cu1 and Cu2, indicating that each have a trigonal-pyramidal geometry (τ_4_ = 1 for a perfect tetra­hedral geometry, = 0 for a perfect square planar geometry and = 0.85 for a perfect trigonal-pyramidal geometry; Yang *et al.*, 2007 ▸).

The coordination environment for each of the Cu^I^ centers includes three bridging di­thiol­ane ligands and one terminal aceto­nitrile ligand. All Cu—S bond lengths are in the range 2.2630 (10)–2.3367 (11) Å, the mean Cu—S bond length of 2.314 (12) Å is quite similar to that in [Cu(1,3-di­thiane)(MeCN)]_*n*_^+^. In addition, the mean Cu—N bond distance matches well with that of [Cu(1,3-di­thiane)(MeCN)]_*n*_ [1.979 (4) *versus* 1.984 (7) Å]. The three di­thiol­ane ligands each have one S atom that is a two-electron donor and one S atom that is a μ_2_-four-electron donor. The Cu⋯Cu separations of *ca* 3.689–3.852 Å are far above the sum of the van der Waals radii of two Cu atoms (2.8 Å), excluding any bonding inter­action. These two bonding modes lead to the formation of a ribbon-like coordination polymer, which runs parallel to the *a* axis, where each copper(I) center is bonded to two μ_2_-S atoms and one μ_1_-S atom (Fig. 2 ▸ and Table 1 ▸).

## Supra­molecular features   

**Figure 3:**
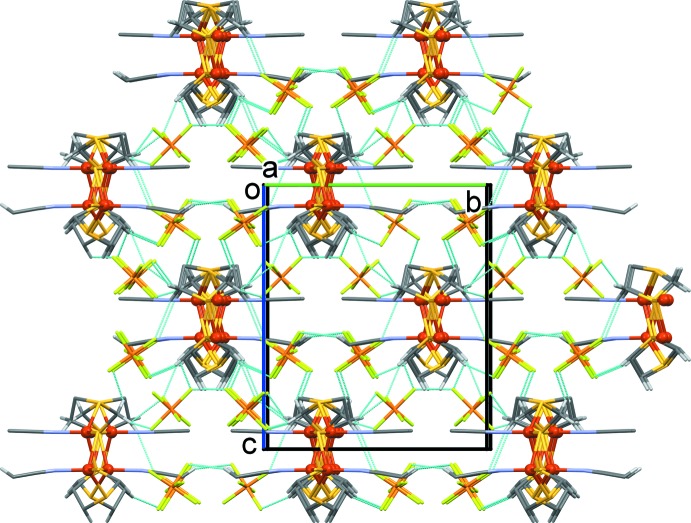
A view along the *a* axis of the crystal packing of the title compound. The C—H⋯F hydrogen bonds (Table 2 ▸) are shown as dashed lines. For clarity, only the H atoms involved in these inter­actions have been included.

The crystal packing of the title compound is illustrated in Fig. 3 ▸, and shows the ribbon-like structures, propagating along the *a*-axis direction, that are linked by a number of C—H⋯F hydrogen bonds, forming a supra­molecular framework (Fig. 3 ▸ and Table 2 ▸).

## Database survey   

Other examples of crystallographically characterized 1,3-di­thiol­ane complexes substituted at the 2-position found in the Cambridge Structural Database (CSD, version 5.40, update August 2019; Groom *et al.*, 2016 ▸) include *catena*-[(μ_5_-1,3-di­thiol­ane-2-carboxyl­ato)(μ_4_-1,3-di­thiol­ane-2-carboxyl­ato)(μ_2_-tri­fluoro­methane­sulfonato-*O*,*O*′)tris­ilver(I)] (CSD refcode FAQIPY; Gondi *et al.*, 2011 ▸), *catena*-[(μ_3_-1,3-di­thiol­ane-2-methanol-*S*,*S*,*S*′)(nitrato-*O*)silver(I)] (HESLUN; Zhang *et al.*, 2006 ▸), chloro­tri­phenyl­phosphine[2,5-bis­(1,3-di­thio­lan-2-yl)phen­yl-*S*]palladium(II) (IVUFEK; Vicente *et al.*, 2004 ▸), *rac*-*trans*-di­chloro­bis­{[2-(1,3-di­thio­lan-2-yl)phen­yl](diphen­yl)phos­phine}ruthenium(II) chloro­form solvate (TUMKOC; Bayly *et al.*, 2009 ▸). Other examples of related 1,3-di­thiane copper(I) coordination polymers have also been reported (Raghuvanshi *et al.*, 2019 ▸).

## Synthesis and crystallization   

**Figure 4:**
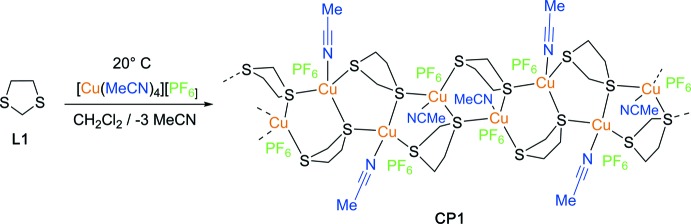
Reaction scheme for the synthesis of the title compound, **CP1**.

The reaction scheme for the synthesis of the title compound is illustrated in Fig. 4 ▸. To a solution of [Cu(MeCN)_4_][PF_6_] (372 mg, 0.1 mmol) in CH_2_Cl_2_ (10 ml) was added an equimolar amount of 1,3-di­thiol­ane (**L1**) *via* a syringe. The solution was stirred at 293 K for 2 h, then layered with Et_2_O (10 ml) and stored in a refrigerator for 2 days. Colourless block-like crystals formed progressively (245 mg, 68% yield).

Elemental analysis calculated for C_10_H_18_Cu_2_F_12_N_2_P_2_S_4_: C, 16.88; H, 2.54; N, 3.94; S, 18.03%. Found: C, 16.44; H, 2.28; N, 3.44; S, 17.81%. IR (ATR; cm^−1^): 2280 *w* (weak) (CN), 835 *vs* (very strong) (PF_6_).

## Refinement   

Crystal data, data collection and structure refinement details are summarized in Table 3 ▸. The C-bound H atoms were included in calculated positions and treated as riding: C—H = 0.98–0.99 Å with *U*_iso_(H) = 1.5*U*_eq_(C-meth­yl) and 1.2*U*_eq_(C) for other H atoms. The structure was refined as a two-component inversion twin; BASF = 0.121 (12). In the final cycles of refinement three reflections were omitted; one was affected by the backstop and two were most disagreeable reflections.

## Supplementary Material

Crystal structure: contains datablock(s) Global, I. DOI: 10.1107/S205698901901627X/su5530sup1.cif

Structure factors: contains datablock(s) I. DOI: 10.1107/S205698901901627X/su5530Isup2.hkl

Supporting information file. DOI: 10.1107/S205698901901627X/su5530Isup3.cdx

CCDC reference: 1969688

Additional supporting information:  crystallographic information; 3D view; checkCIF report

## Figures and Tables

**Table 1:** Selected geometric parameters (Å, °)

Cu1—N1	1.973 (3)	Cu2—N2	1.980 (3)
Cu1—S1	2.2630 (10)	Cu2—S3	2.2886 (11)
Cu1—S2^i^	2.3305 (9)	Cu2—S4^i^	2.3281 (9)
Cu1—S4^i^	2.3367 (11)	Cu2—S2	2.3357 (11)
			
N1—Cu1—S1	119.47 (11)	N2—Cu2—S4^i^	99.29 (11)
N1—Cu1—S2^i^	99.97 (9)	S3—Cu2—S4^i^	118.69 (4)
S1—Cu1—S2^i^	115.68 (4)	N2—Cu2—S2	106.03 (13)
N1—Cu1—S4^i^	105.68 (12)	S3—Cu2—S2	115.99 (4)
S1—Cu1—S4^i^	110.65 (4)	S4^i^—Cu2—S2	102.03 (4)
S2^i^—Cu1—S4^i^	103.69 (4)	Cu1^ii^—S2—Cu2	111.28 (4)
N2—Cu2—S3	112.75 (12)	Cu2^ii^—S4—Cu1^ii^	104.54 (4)

Symmetry codes: (i) 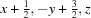; (ii) 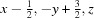.

**Table 2:** Hydrogen-bond geometry (Å, °)

*D*—H⋯*A*	*D*—H	H⋯*A*	*D*⋯*A*	*D*—H⋯*A*
C1—H1*A*⋯F9^iii^	0.99	2.55	3.264 (4)	129
C1—H1*A*⋯F12^iii^	0.99	2.40	3.277 (5)	147
C2—H2*A*⋯F9^iii^	0.99	2.50	3.287 (5)	136
C3—H3*B*⋯F4^ii^	0.99	2.42	3.376 (5)	161
C5—H5*C*⋯F6^iv^	0.98	2.54	3.426 (6)	151
C8—H8*A*⋯F11	0.99	2.34	3.186 (5)	143
C8—H8*B*⋯F2^v^	0.99	2.46	3.323 (5)	145
C10—H10*A*⋯F7^ii^	0.99	2.31	3.221 (5)	152
C10—H10*B*⋯F1^ii^	0.99	2.48	3.264 (5)	136

Symmetry codes: (ii) 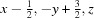; (iii) 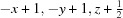; (iv) 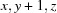; (v) 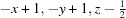.

**Table 3:** Experimental details

Crystal data	
Chemical formula	[Cu_2_(C_2_H_3_N)_2_(C_3_H_6_S_2_)_2_] (PF_6_)_2_
*M*_r_	711.52
Crystal system, space group	Orthorhombic, *P* *n* *a*2_1_
Temperature (K)	105
*a*, *b*, *c* (Å)	11.8409 (9), 12.9273 (9), 15.2921 (11)
*V* (Å^3^)	2340.8 (3)
*Z*	4
Radiation type	Mo *K*α
μ (mm^−1^)	2.41
Crystal size (mm)	0.33 × 0.32 × 0.27

Data collection	
Diffractometer	Bruker D8 VENTURE area detector
Absorption correction	Multi-scan (*TWINABS*; Bruker, 2016 ▸)
*T*_min_, *T*_max_	0.608, 0.746
No. of measured, independent and observed [*I* > 2σ(*I*)] reflections	40393, 8122, 7092
*R*_int_	0.040
(sin θ/λ)_max_ (Å^−1^)	0.769

Refinement	
*R*[*F* ^2^ > 2σ(*F* ^2^)], *wR*(*F* ^2^), *S*	0.034, 0.080, 1.03
No. of reflections	8122
No. of parameters	293
No. of restraints	1
H-atom treatment	H-atom parameters constrained
Δρ_max_, Δρ_min_ (e Å^−3^)	0.93, −0.73

Computer programs: *APEX2* and *SAINT* (Bruker, 2016 ▸), *SHELXT* (Sheldrick, 2015*a* ▸), *SHELXL* (Sheldrick, 2015*b* ▸), *OLEX2* (Dolomanov *et al.*, 2009 ▸), *Mercury* (Macrae *et al.*, 2008 ▸), *PLATON* (Spek, 2009 ▸) and *publCIF* (Westrip, 2010 ▸).

## supplementary crystallographic information

### Crystal data

**Table:**

[Cu_2_(C_2_H_3_N)_2_(C_3_H_6_S_2_)_2_](PF_6_)_2_	*D*_x_ = 2.019 Mg m^−^^3^
*M**_r_* = 711.52	Mo *K*α radiation, λ = 0.71073 Å
Orthorhombic, *P**n**a*2_1_	Cell parameters from 9565 reflections
*a* = 11.8409 (9) Å	θ = 2.7–31.8°
*b* = 12.9273 (9) Å	µ = 2.41 mm^−^^1^
*c* = 15.2921 (11) Å	*T* = 105 K
*V* = 2340.8 (3) Å^3^	Block, colourless
*Z* = 4	0.33 × 0.32 × 0.27 mm
*F*(000) = 1408	

### Data collection

**Table:**

Bruker D8 VENTURE area detector diffractometer	8122 independent reflections
Radiation source: microfocus sealed X-ray tube, Incoatec Iµs	7092 reflections with *I* > 2σ(*I*)
HELIOS mirror optics monochromator	*R*_int_ = 0.040
Detector resolution: 10.4167 pixels mm^-1^	θ_max_ = 33.1°, θ_min_ = 2.3°
ω and φ scans	*h* = −17→17
Absorption correction: multi-scan (*TWINABS*; Bruker, 2016)	*k* = −19→18
*T*_min_ = 0.608, *T*_max_ = 0.746	*l* = −21→23
40393 measured reflections	

### Refinement

**Table:**

Refinement on *F*^2^	Hydrogen site location: inferred from neighbouring sites
Least-squares matrix: full	H-atom parameters constrained
*R*[*F*^2^ > 2σ(*F*^2^)] = 0.034	*w* = 1/[σ^2^(*F*_o_^2^) + (0.0388*P*)^2^ + 1.7169*P*] where *P* = (*F*_o_^2^ + 2*F*_c_^2^)/3
*wR*(*F*^2^) = 0.080	(Δ/σ)_max_ = 0.001
*S* = 1.03	Δρ_max_ = 0.93 e Å^−^^3^
8122 reflections	Δρ_min_ = −0.73 e Å^−^^3^
293 parameters	Extinction correction: (SHELXL-2018/3; Sheldrick, 2015b), Fc^*^=kFc[1+0.001xFc^2^λ^3^/sin(2θ)]^-1/4^
1 restraint	Extinction coefficient: 0.0012 (3)
Primary atom site location: dual	Absolute structure: Refined as an inversion twin.
Secondary atom site location: difference Fourier map	Absolute structure parameter: 0.115 (11)

### Special details

**Table:**

Geometry. All esds (except the esd in the dihedral angle between two l.s. planes) are estimated using the full covariance matrix. The cell esds are taken into account individually in the estimation of esds in distances, angles and torsion angles; correlations between esds in cell parameters are only used when they are defined by crystal symmetry. An approximate (isotropic) treatment of cell esds is used for estimating esds involving l.s. planes.
Refinement. Refined as a two-component inversion twin

### Fractional atomic coordinates and isotropic or equivalent isotropic displacement parameters (Å^2^)

**Table:**

	*x*	*y*	*z*	*U*_iso_*/*U*_eq_	
Cu1	0.84315 (3)	0.78973 (3)	0.57850 (3)	0.01740 (10)	
Cu2	0.59217 (4)	0.80128 (3)	0.43586 (3)	0.01907 (10)	
S1	0.73437 (7)	0.71169 (7)	0.68074 (6)	0.01758 (17)	
S2	0.53256 (7)	0.75921 (6)	0.57705 (6)	0.01552 (15)	
S3	0.47858 (7)	0.74560 (8)	0.32430 (7)	0.02113 (18)	
S4	0.28017 (7)	0.75139 (6)	0.43792 (6)	0.01688 (16)	
N1	0.8599 (3)	0.9416 (2)	0.5802 (3)	0.0218 (6)	
N2	0.6146 (3)	0.9531 (3)	0.4352 (3)	0.0271 (7)	
C1	0.6181 (3)	0.6541 (3)	0.6216 (3)	0.0176 (6)	
H1A	0.572111	0.610665	0.661277	0.021*	
H1B	0.646981	0.609859	0.573648	0.021*	
C2	0.6454 (3)	0.8120 (3)	0.7273 (3)	0.0216 (7)	
H2A	0.595280	0.781936	0.772430	0.026*	
H2B	0.692894	0.865649	0.755319	0.026*	
C3	0.5748 (3)	0.8604 (3)	0.6548 (3)	0.0229 (8)	
H3A	0.619383	0.914115	0.624196	0.027*	
H3B	0.506878	0.893506	0.680033	0.027*	
C4	0.8973 (3)	1.0220 (3)	0.5899 (3)	0.0233 (7)	
C5	0.9471 (4)	1.1236 (3)	0.6047 (3)	0.0337 (10)	
H5A	1.026569	1.122572	0.586966	0.051*	
H5B	0.941772	1.141126	0.666932	0.051*	
H5C	0.906273	1.175400	0.570202	0.051*	
C6	0.6404 (4)	1.0373 (4)	0.4332 (4)	0.0384 (10)	
C7	0.6757 (8)	1.1462 (5)	0.4299 (6)	0.081 (3)	
H7A	0.631809	1.182650	0.385163	0.122*	
H7B	0.756221	1.150048	0.415362	0.122*	
H7C	0.662709	1.178524	0.486982	0.122*	
C8	0.3636 (3)	0.6684 (3)	0.3662 (3)	0.0212 (7)	
H8A	0.393246	0.608356	0.399149	0.025*	
H8B	0.316389	0.642406	0.317440	0.025*	
C9	0.3864 (3)	0.8562 (3)	0.3071 (3)	0.0250 (8)	
H9A	0.431480	0.917698	0.290743	0.030*	
H9B	0.332220	0.841488	0.259437	0.030*	
C10	0.3233 (3)	0.8761 (3)	0.3924 (3)	0.0241 (8)	
H10A	0.256090	0.919716	0.381185	0.029*	
H10B	0.372927	0.912903	0.434195	0.029*	
P1	0.83561 (9)	0.41114 (8)	0.64167 (8)	0.0243 (2)	
F1	0.8514 (2)	0.5141 (2)	0.5834 (2)	0.0352 (6)	
F2	0.8195 (2)	0.3098 (2)	0.7025 (2)	0.0381 (7)	
F3	0.9652 (2)	0.3807 (2)	0.6263 (2)	0.0400 (6)	
F4	0.8733 (3)	0.4757 (2)	0.72667 (18)	0.0373 (6)	
F5	0.7068 (2)	0.4413 (2)	0.6591 (3)	0.0478 (8)	
F6	0.8011 (3)	0.3459 (3)	0.5585 (2)	0.0564 (10)	
P2	0.56386 (9)	0.42170 (8)	0.34899 (7)	0.0242 (2)	
F7	0.6297 (4)	0.5004 (3)	0.2875 (2)	0.0639 (11)	
F8	0.4947 (4)	0.3435 (3)	0.4073 (2)	0.0765 (14)	
F9	0.5737 (2)	0.3338 (2)	0.27453 (18)	0.0295 (5)	
F10	0.6798 (2)	0.3850 (2)	0.3895 (2)	0.0402 (7)	
F11	0.5557 (2)	0.5097 (2)	0.4233 (2)	0.0394 (7)	
F12	0.4494 (3)	0.4601 (3)	0.3056 (3)	0.0836 (17)	

### Atomic displacement parameters (Å^2^)

**Table:**

	*U*^11^	*U*^22^	*U*^33^	*U*^12^	*U*^13^	*U*^23^
Cu1	0.01103 (17)	0.02016 (19)	0.0210 (2)	−0.00064 (14)	0.00107 (16)	−0.00060 (19)
Cu2	0.01171 (18)	0.0229 (2)	0.0226 (2)	−0.00045 (15)	0.00019 (17)	0.00155 (19)
S1	0.0107 (3)	0.0221 (4)	0.0200 (4)	0.0000 (3)	−0.0009 (3)	0.0023 (3)
S2	0.0092 (3)	0.0186 (3)	0.0188 (4)	0.0002 (3)	0.0004 (3)	0.0008 (4)
S3	0.0130 (4)	0.0303 (4)	0.0201 (4)	0.0001 (3)	0.0025 (3)	−0.0025 (4)
S4	0.0098 (3)	0.0221 (4)	0.0187 (4)	0.0007 (3)	0.0008 (3)	0.0005 (4)
N1	0.0211 (14)	0.0210 (13)	0.0232 (15)	0.0036 (11)	0.0000 (14)	−0.0005 (14)
N2	0.0294 (17)	0.0244 (14)	0.0275 (16)	−0.0013 (13)	−0.0028 (17)	0.0032 (16)
C1	0.0109 (13)	0.0188 (15)	0.0232 (17)	0.0004 (12)	−0.0012 (13)	0.0041 (13)
C2	0.0162 (16)	0.0276 (17)	0.0210 (18)	−0.0007 (13)	0.0018 (13)	−0.0030 (15)
C3	0.0167 (16)	0.0221 (16)	0.030 (2)	0.0020 (13)	−0.0030 (14)	−0.0058 (15)
C4	0.0253 (18)	0.0239 (17)	0.0208 (18)	0.0042 (14)	−0.0024 (15)	−0.0012 (15)
C5	0.044 (3)	0.0210 (18)	0.036 (2)	−0.0023 (17)	−0.010 (2)	−0.0038 (17)
C6	0.050 (3)	0.030 (2)	0.036 (2)	−0.0020 (19)	−0.009 (2)	0.006 (2)
C7	0.115 (7)	0.031 (3)	0.097 (6)	−0.017 (3)	−0.028 (6)	0.022 (4)
C8	0.0131 (15)	0.0248 (17)	0.026 (2)	−0.0002 (13)	0.0027 (13)	−0.0048 (15)
C9	0.0189 (17)	0.032 (2)	0.0241 (19)	−0.0009 (14)	−0.0017 (14)	0.0094 (16)
C10	0.0189 (17)	0.0233 (17)	0.030 (2)	0.0052 (14)	0.0044 (15)	0.0058 (15)
P1	0.0237 (5)	0.0215 (4)	0.0276 (5)	0.0015 (4)	−0.0041 (4)	0.0034 (4)
F1	0.0441 (15)	0.0313 (12)	0.0302 (13)	0.0015 (11)	−0.0016 (13)	0.0093 (12)
F2	0.0352 (14)	0.0288 (13)	0.0505 (18)	0.0056 (11)	0.0069 (13)	0.0147 (12)
F3	0.0305 (13)	0.0460 (15)	0.0434 (16)	0.0103 (12)	0.0071 (12)	0.0068 (14)
F4	0.0461 (16)	0.0389 (14)	0.0269 (13)	−0.0004 (13)	−0.0021 (13)	−0.0024 (11)
F5	0.0238 (13)	0.0352 (14)	0.084 (3)	0.0063 (11)	−0.0003 (14)	0.0167 (16)
F6	0.080 (3)	0.0400 (16)	0.049 (2)	0.0039 (16)	−0.0306 (18)	−0.0073 (14)
P2	0.0207 (4)	0.0254 (5)	0.0267 (5)	0.0014 (4)	−0.0027 (4)	−0.0060 (4)
F7	0.125 (3)	0.0390 (17)	0.0273 (15)	−0.034 (2)	−0.010 (2)	0.0043 (13)
F8	0.089 (3)	0.086 (3)	0.054 (2)	−0.059 (2)	0.040 (2)	−0.024 (2)
F9	0.0261 (12)	0.0282 (11)	0.0341 (14)	0.0008 (10)	−0.0020 (10)	−0.0097 (11)
F10	0.0383 (15)	0.0422 (15)	0.0400 (16)	0.0129 (13)	−0.0168 (13)	−0.0089 (13)
F11	0.0354 (14)	0.0431 (14)	0.0397 (17)	0.0112 (12)	−0.0089 (12)	−0.0241 (13)
F12	0.061 (2)	0.093 (3)	0.097 (3)	0.052 (2)	−0.053 (2)	−0.069 (3)

### Geometric parameters (Å, º)

**Table:**

Cu1—N1	1.973 (3)	C5—H5A	0.9800
Cu1—S1	2.2630 (10)	C5—H5B	0.9800
Cu1—S2^i^	2.3305 (9)	C5—H5C	0.9800
Cu1—S4^i^	2.3367 (11)	C6—C7	1.469 (7)
Cu2—N2	1.980 (3)	C7—H7A	0.9800
Cu2—S3	2.2886 (11)	C7—H7B	0.9800
Cu2—S4^i^	2.3281 (9)	C7—H7C	0.9800
Cu2—S2	2.3357 (11)	C8—H8A	0.9900
S1—C1	1.808 (4)	C8—H8B	0.9900
S1—C2	1.817 (4)	C9—C10	1.524 (6)
S2—C1	1.827 (4)	C9—H9A	0.9900
S2—C3	1.836 (4)	C9—H9B	0.9900
S3—C8	1.806 (4)	C10—H10A	0.9900
S3—C9	1.817 (4)	C10—H10B	0.9900
S4—C8	1.825 (4)	P1—F6	1.579 (3)
S4—C10	1.830 (4)	P1—F5	1.597 (3)
N1—C4	1.140 (5)	P1—F3	1.602 (3)
N2—C6	1.132 (6)	P1—F4	1.608 (3)
C1—H1A	0.9900	P1—F1	1.613 (3)
C1—H1B	0.9900	P1—F2	1.617 (3)
C2—C3	1.523 (6)	P2—F8	1.577 (4)
C2—H2A	0.9900	P2—F10	1.579 (3)
C2—H2B	0.9900	P2—F12	1.588 (3)
C3—H3A	0.9900	P2—F7	1.590 (4)
C3—H3B	0.9900	P2—F11	1.611 (3)
C4—C5	1.456 (6)	P2—F9	1.613 (3)
			
N1—Cu1—S1	119.47 (11)	H5B—C5—H5C	109.5
N1—Cu1—S2^i^	99.97 (9)	N2—C6—C7	179.0 (7)
S1—Cu1—S2^i^	115.68 (4)	C6—C7—H7A	109.5
N1—Cu1—S4^i^	105.68 (12)	C6—C7—H7B	109.5
S1—Cu1—S4^i^	110.65 (4)	H7A—C7—H7B	109.5
S2^i^—Cu1—S4^i^	103.69 (4)	C6—C7—H7C	109.5
N2—Cu2—S3	112.75 (12)	H7A—C7—H7C	109.5
N2—Cu2—S4^i^	99.29 (11)	H7B—C7—H7C	109.5
S3—Cu2—S4^i^	118.69 (4)	S3—C8—S4	107.2 (2)
N2—Cu2—S2	106.03 (13)	S3—C8—H8A	110.3
S3—Cu2—S2	115.99 (4)	S4—C8—H8A	110.3
S4^i^—Cu2—S2	102.03 (4)	S3—C8—H8B	110.3
C1—S1—C2	92.80 (17)	S4—C8—H8B	110.3
C1—S1—Cu1	105.74 (13)	H8A—C8—H8B	108.5
C2—S1—Cu1	106.41 (13)	C10—C9—S3	107.7 (3)
C1—S2—C3	97.91 (17)	C10—C9—H9A	110.2
C1—S2—Cu1^ii^	109.15 (11)	S3—C9—H9A	110.2
C3—S2—Cu1^ii^	116.70 (13)	C10—C9—H9B	110.2
C1—S2—Cu2	110.50 (13)	S3—C9—H9B	110.2
C3—S2—Cu2	110.50 (14)	H9A—C9—H9B	108.5
Cu1^ii^—S2—Cu2	111.28 (4)	C9—C10—S4	108.2 (3)
C8—S3—C9	91.90 (18)	C9—C10—H10A	110.0
C8—S3—Cu2	110.66 (14)	S4—C10—H10A	110.0
C9—S3—Cu2	102.31 (14)	C9—C10—H10B	110.0
C8—S4—C10	97.95 (18)	S4—C10—H10B	110.0
C8—S4—Cu2^ii^	109.72 (13)	H10A—C10—H10B	108.4
C10—S4—Cu2^ii^	121.31 (13)	F6—P1—F5	91.0 (2)
C8—S4—Cu1^ii^	104.29 (13)	F6—P1—F3	89.9 (2)
C10—S4—Cu1^ii^	117.50 (15)	F5—P1—F3	178.8 (2)
Cu2^ii^—S4—Cu1^ii^	104.54 (4)	F6—P1—F4	178.58 (19)
C4—N1—Cu1	161.5 (3)	F5—P1—F4	90.19 (18)
C6—N2—Cu2	172.0 (4)	F3—P1—F4	88.87 (17)
S1—C1—S2	107.58 (19)	F6—P1—F1	91.48 (18)
S1—C1—H1A	110.2	F5—P1—F1	90.12 (16)
S2—C1—H1A	110.2	F3—P1—F1	90.55 (16)
S1—C1—H1B	110.2	F4—P1—F1	89.24 (16)
S2—C1—H1B	110.2	F6—P1—F2	89.98 (19)
H1A—C1—H1B	108.5	F5—P1—F2	89.37 (16)
C3—C2—S1	109.0 (3)	F3—P1—F2	89.94 (15)
C3—C2—H2A	109.9	F4—P1—F2	89.31 (17)
S1—C2—H2A	109.9	F1—P1—F2	178.46 (18)
C3—C2—H2B	109.9	F8—P2—F10	92.1 (2)
S1—C2—H2B	109.9	F8—P2—F12	89.6 (3)
H2A—C2—H2B	108.3	F10—P2—F12	178.1 (3)
C2—C3—S2	109.2 (3)	F8—P2—F7	177.8 (2)
C2—C3—H3A	109.8	F10—P2—F7	89.9 (2)
S2—C3—H3A	109.8	F12—P2—F7	88.4 (3)
C2—C3—H3B	109.8	F8—P2—F11	91.27 (19)
S2—C3—H3B	109.8	F10—P2—F11	89.26 (15)
H3A—C3—H3B	108.3	F12—P2—F11	91.32 (16)
N1—C4—C5	178.2 (5)	F7—P2—F11	89.70 (18)
C4—C5—H5A	109.5	F8—P2—F9	89.15 (17)
C4—C5—H5B	109.5	F10—P2—F9	90.17 (15)
H5A—C5—H5B	109.5	F12—P2—F9	89.25 (16)
C4—C5—H5C	109.5	F7—P2—F9	89.90 (17)
H5A—C5—H5C	109.5	F11—P2—F9	179.30 (16)
			
C2—S1—C1—S2	40.7 (2)	C9—S3—C8—S4	−41.9 (2)
Cu1—S1—C1—S2	−67.21 (18)	Cu2—S3—C8—S4	62.0 (2)
C3—S2—C1—S1	−23.4 (2)	C10—S4—C8—S3	22.5 (2)
Cu1^ii^—S2—C1—S1	−145.34 (13)	Cu2^ii^—S4—C8—S3	149.92 (14)
Cu2—S2—C1—S1	91.98 (18)	Cu1^ii^—S4—C8—S3	−98.58 (18)
C1—S1—C2—C3	−48.2 (3)	C8—S3—C9—C10	51.8 (3)
Cu1—S1—C2—C3	59.1 (3)	Cu2—S3—C9—C10	−59.9 (3)
S1—C2—C3—S2	37.2 (3)	S3—C9—C10—S4	−41.7 (3)
C1—S2—C3—C2	−8.0 (3)	C8—S4—C10—C9	11.4 (3)
Cu1^ii^—S2—C3—C2	108.1 (2)	Cu2^ii^—S4—C10—C9	−107.6 (2)
Cu2—S2—C3—C2	−123.5 (2)	Cu1^ii^—S4—C10—C9	122.1 (3)

Symmetry codes: (i) *x*+1/2, −*y*+3/2, *z*; (ii) *x*−1/2, −*y*+3/2, *z*.

### Hydrogen-bond geometry (Å, º)

**Table:**

*D*—H···*A*	*D*—H	H···*A*	*D*···*A*	*D*—H···*A*
C1—H1*A*···F9^iii^	0.99	2.55	3.264 (4)	129
C1—H1*A*···F12^iii^	0.99	2.40	3.277 (5)	147
C2—H2*A*···F9^iii^	0.99	2.50	3.287 (5)	136
C3—H3*B*···F4^ii^	0.99	2.42	3.376 (5)	161
C5—H5*C*···F6^iv^	0.98	2.54	3.426 (6)	151
C8—H8*A*···F11	0.99	2.34	3.186 (5)	143
C8—H8*B*···F2^v^	0.99	2.46	3.323 (5)	145
C10—H10*A*···F7^ii^	0.99	2.31	3.221 (5)	152
C10—H10*B*···F1^ii^	0.99	2.48	3.264 (5)	136

Symmetry codes: (ii) *x*−1/2, −*y*+3/2, *z*; (iii) −*x*+1, −*y*+1, *z*+1/2; (iv) *x*, *y*+1, *z*; (v) −*x*+1, −*y*+1, *z*−1/2.
